# Liberalizing consent - Supreme Court's preference for ‘real consent’ over ‘informed consent’

**Published:** 2008-08

**Authors:** ATM Rangaramanujam

Consent is a major source of dispute between doctors and patients, not just in India but all over the world. There are two major schools of thought which govern the law of consent in medicine. One is the doctrine of ‘informed consent,’ according to which it is the doctor's responsibility to disclose the necessary information to the patient to secure consent. Under the doctrine of ‘real consent’ the doctor must warn his patient of the risks inherent in the recommended treatment and the terms of giving such warning must be in accordance with the practice accepted at that time as considered proper by a responsible body of medical opinion. This is Bolam's law or ‘Real Consent.’

In the present case *(Ed note: Samira Kohli, see below)*, the Supreme Court has preferred ‘real consent’ followed in the UK over ‘informed consent’ followed in America, in the following terms:

‘We are of the view that to nurture the doctor-patient relationship on the basis of trust, the extent and nature of information required to be given by doctors should continue to be governed by the Bolam test rather than the ‘reasonably prudential patient’ test evolved in Canterbury. It is for the doctor to decide, with reference to the condition of the patient, the nature of illness, and the prevailing established practices, how much information regarding risks and consequences should be given to the patients, and how they should be couched, having the best interests of the patient. A doctor cannot be held negligent either in regard to diagnosis or treatment or in disclosing the risks involved in a particular surgical procedure or treatment, if the doctor has acted with normal care, in accordance with recognized practices accepted as proper by a responsible body of medical men skilled in that particular field, even though there may be a body of opinion that takes a contrary view.’

The elements of real consent as prescribed in the present judgment are:

- The patient gives it voluntarily without any coercion
- The patient has the capacity and competence to give consent
- The patient has an adequate level of information about the nature of the procedure to which he is consenting

When a doctor is specifically questioned by the patient about the risks involved in a particular treatment proposed, the doctor's duty is to answer truthfully and as fully as the patient requires. Remote risk of harm (referred to as 1-2% risk) need not be disclosed, but if the risk of harm is substantial (referred to as 10% risk), it may have to be disclosed.

However, the most important part of this judgment, the ‘heart,’ is the reason given by the Court for preferring real consent over informed consent.

In India, majority of citizens requiring medical care and treatment fall below the poverty line. Most of them are illiterate or semi-literate. They cannot comprehend medical terms, concepts, and treatment procedures. They cannot understand the functions of various organs or the effect of removal of such organs. They do not have access to effective but costly diagnostic procedures. Poor patients lying in the corridors of hospitals after admission for want of beds or patients waiting for days on the roadside for an admission or a mere examination is a common sight. For them, any treatment with reference to rough and ready diagnosis based on their outward symptoms and the doctor's experience or intuition is acceptable and welcome so long as it is free or cheap; and whatever the doctor decides as being in their interest, is usually unquestioningly accepted. They are passive, ignorant, and uninvolved in treatment procedures.

‘There is a need to keep the cost of treatment within affordable limits. Bringing in the American concepts and standards of treatment procedures and disclosure of risks, consequences and choices will inevitably bring in the higher cost structure of American medical care. Patients in India cannot afford them.’

Applying real consent in real-life situations would mean that the doctor's discretion in disclosing relevant information and risks will be greatly enhanced. Law of consent will become more refined once the lower courts, especially consumer courts, apply this judgment to different facts and circumstances. As courts start prescribing subjectively the various do's and don'ts, the real effect of this judgment will become evident, relieving the doctors from stringent norms of taking patient's consent.

This judgment ought to be welcomed by the medical fraternity as the norms for taking consent have been liberalized, summarized, and specifically laid down by the apex court of this country. It must also be welcomed by patients, especially the common man, because the Supreme Court has taken note of the high and rising cost of health care and the present judgment is a small positive step aimed at addressing this situation.

In 1996, in V.P. Shantha's case, a plea was raised by doctors that if they come within the purview of the Consumer Protection Act, the courts will be flooded with cases of medical negligence and it will become impossible for them to discharge their professional duties. But the Supreme Court rejected this argument outright.

In 2005, in Jacob Mathew's case, the Supreme Court took note of the rising number of cases in medical negligence and its adverse effect on doctors. Thanks to the various privileges that have been given exclusively to doctors by this judgment, arresting a doctor for medical negligence has become nearly impossible in this country.

In 2008, in Samira Kohli's case (present case) the Supreme Court has liberalized the procedure of taking patient's consent. Hopefully this will make the medical fraternity happy. But the onus on the medical fraternity to self-regulate has simultaneously increased manifold. If they fail, the liberties offered to them may be withdrawn and the law may become more stringent. This reverse process has already started in many Western countries, including England. The Supreme Court, in the present case, has aptly warned the doctors.

‘We have, however, consciously preferred the ‘real consent’ concept evolved in Bolam and Sidaway in preference to the ‘reasonably prudent patient test’ of Canterbury, having regard to the ground realities in medical and health care in India. But if medical practitioners and private hospitals become more and more commercialized, and if there is a corresponding increase in the awareness of patient's rights among the public, inevitably, a day may come when we may have to move towards Canterbury. But not for the present.’

## Summary of the law of consent

Excerpts of the Supreme Court's judgment:

‘We may now summarize principles relating to consent as follows:

- A doctor has to seek and secure the consent of the patient before commencing a ‘treatment’ (the term ‘treatment’ includes surgery also). The consent so obtained should be real and valid, which means that: the patient should have the capacity and competence to consent; his consent should be voluntary; and his consent should be on the basis of adequate information concerning the nature of the treatment procedure, so that he knows what he is consenting to.
- The ‘adequate information’ to be furnished by the doctor (or a member of his team) who treats the patient, should enable the patient to make a balanced judgment as to whether he should submit to the particular treatment or not. This means that the doctor should disclose (a) the nature and procedure of the treatment and its purpose, benefits, and effect; (b) alternatives, if any, available; (c) an outline of the substantial risks; and (d) adverse consequences of refusing treatment. But there is no need to explain remote or theoretical risks involved, which may frighten or confuse a patient and result in refusal of consent for the necessary treatment. Similarly, there is no need to explain the remote or theoretical risks of refusal to take treatment, which may persuade a patient to undergo a fanciful or unnecessary treatment. A balance should be achieved between the need for disclosing necessary and adequate information and at the same time avoid the possibility of the patient being deterred from agreeing to a necessary treatment or offering to undergo an unnecessary treatment.
- Consent given only for a diagnostic procedure, cannot be considered as consent for therapeutic treatment. Consent given for a specific treatment procedure will not be valid for conducting some other treatment procedure. The fact that the unauthorized additional surgery is beneficial to the patient, or that it would save considerable time and expense to the patient, or would relieve the patient from pain and suffering in future, are not grounds of defense in an action in tort for negligence or assault and battery. The only exception to this rule is where the additional procedure, though unauthorized, is necessary in order to save the life or preserve the health of the patient and it would be unreasonable to delay such unauthorized procedure until patient regains consciousness and takes a decision.
- There can be a common consent for diagnostic and operative procedures where they are contemplated. There can also be a common consent for a particular surgical procedure and an additional or further procedure that may become necessary during the course of surgery.
- The nature and extent of information to be furnished by the doctor to the patient to secure the consent need not be of the stringent and high degree mentioned in Canterbury but should be of the extent which is accepted as normal and proper by a body of medical men skilled and experienced in the particular field. It will depend upon the physical and mental condition of the patient, the nature of treatment, and the risk and consequences attached to the treatment.’

